# Dynamic Perfusion Computed Tomography for the Assessment of Concomitant Coronary Artery Disease in Patients with a History of Percutaneous Transluminal Angioplasty for Chronic Limb-Threatening Ischemia—A Pilot Study

**DOI:** 10.3390/jcdd10110443

**Published:** 2023-10-25

**Authors:** Ferenc T. Nagy, Dorottya Olajos, Borbála Vattay, Sarolta Borzsák, Melinda Boussoussou, Mónika Deák, Milán Vecsey-Nagy, Barbara Sipos, Ádám L. Jermendy, Gábor G. Tóth, Balázs Nemes, Béla Merkely, Tamás Szili-Török, Zoltán Ruzsa, Bálint Szilveszter

**Affiliations:** 1 Division of Invasive Cardiology, Department of Internal Medicine, University of Szeged, 6725 Szeged, Hungary; nagy.ferenc.1@med.u-szeged.hu (F.T.N.); olajosd@gmail.com (D.O.);; 2 Heart and Vascular Center, Semmelweis University, Határőr Str. 18, 1122 Budapest, Hungary; 3 Bács-Kiskun County Hospital, 6725 Kecskemét, Hungary; 4 Graz University Heart Center Graz, Medical University of Graz, 8036 Graz, Austria

**Keywords:** chronic limb-threatening ischemia (CLTI), regadenoson stress dynamic perfusion computed tomography (DPCT)

## Abstract

Background: Chronic limb-threatening ischemia (CLTI) is associated with high rates of long-term cardiovascular mortality. Exercise stress testing to detect obstructive coronary artery disease (CAD) can be difficult in this subset of patients due to inability to undergo exercise testing, presence of balanced ischemia and severe coronary artery calcification (CAC). Aim: To test the feasibility of regadenoson stress dynamic perfusion computed tomography (DPCT) in CLTI patients. Methods: Between 2018 and 2023, coronary computed tomography angiography (CTA) and, in the case of a calcium score higher than 400, DPCT, were performed in 25 CLTI patients with a history of endovascular revascularization. Results: Of the 25 patients, 19 had a calcium score higher than 400, requiring DPCT image acquisition. Obstructive CAD could be ruled out in 10 of the 25 patients. Of the 15 CTA/DPCT+ patients, 13 proceeded to coronary angiography (CAG). Revascularization was necessary in all 13 patients. In these 13 patients, vessel-based sensitivity and specificity of coronary CTA/DPCT as compared to invasive evaluation was 75%, respectively. At follow-up (27 ± 21 months) there was no statistically significant difference in all-cause mortality between CTA/DPCT- positive and -negative patients (*p* = 0.065). Conclusions: Despite a high prevalence of severe CAC, coronary CTA complemented by DPCT may be a feasible method to detect obstructive and functionally significant CAD in CLTI patients.

## 1. Introduction

Lower-extremity peripheral artery disease (PAD) affects >230 million adults worldwide [1]. Chronic limb-threatening ischemia (CLTI) in an end-stage manifestation of PAD is characterized by chronic inadequate tissue perfusion at rest [2]. Treatment for CLTI includes medical therapy to reduce cardiovascular risk, revascularization (surgical or endovascular) to improve limb perfusion, and local care to control infection and improve wound healing [3]. In spite of correct revascularization and adequate medical treatment, the long-term prognosis for CLTI patients remains poor [4]. Reported two-year mortality rates are high, ranging approximately between 20 and 30% depending on the initial study population [5,6,7]. Aside from infectious disease, a major driver of mortality, accounting for up to 30% of deaths, is concomitant cardiovascular disease (CVD) [7]. Elevated CVD risk in PAD has been shown to be partially attributed to an abundance of shared conventional risk factors, such as diabetes, smoking, dyslipidemia, obesity, or hypertension [1]. Moreover, for any given level of CVD risk factors, PAD is also independently related to future CVD events and mortality [8].

Screening for concomitant CVD, including coronary artery disease (CAD), is hampered by several issues in CLTI patients. Angina may be atypical or completely missing due to limited movement capability, poor general state, and diabetes. The choice of non-invasive diagnostic test can also be difficult due to inability to undergo exercise testing and the presence of balanced ischemia and of diffuse severe coronary artery calcification [9]. Computed tomography angiography (CTA) has emerged as the preferred diagnostic test for patients with a low-to-intermediate likelihood of having obstructive CAD [10]. Coronary CTA alone, however, cannot determine the functional severity of lesions, and thus additional invasive or non-invasive testing is often necessary. Furthermore, high levels of coronary artery calcification challenge the interpretation of coronary CTA and may lead to overestimation of the stenosis degree [11]. Dynamic perfusion computer tomography (DPCT) is a technique that uses serial CT imaging to measure the inflow of contrast medium into the myocardium to calculate absolute measures of myocardial perfusion [12]. The combination of coronary CTA with DPCT allows for not only anatomical but also functional non-invasive testing for CAD.

In this study, we tested whether screening for obstructive CAD is feasible using CTA complemented with DPCT in patients with a history of percutaneous revascularization because of CLTI.

## 2. Materials and Methods

### 2.1. Study Population and Protocol

Participants for this multicenter registry were recruited from patients who underwent successful percutaneous transluminal angioplasty (PTA) because of CLTI at one of the following three centers participating in the study: (1) Department of Invasive Cardiology, Bács-Kiskun County Hospital; (2) Division of Invasive Cardiology, Department of Internal Medicine, University of Szeged; (3) Heart and Vascular Centre, Faculty of Medicine, Semmelweis University. CLTI was defined as ischemic pain in the foot while a person is at rest, with pain lasting two or more weeks, non-healing wounds, or gangrene that are attributable to objectively proven arterial occlusive disease [13]. The decision to proceed to PTA instead of vascular surgery or conservative management was made beforehand by the vascular team. Exclusion criteria were as follows: (1) relevant coronary artery disease already ruled out by prior examinations; (2) history of coronary artery revascularization; (3) severe valvular disease, heart failure, angina status (CCS III-IV), or ACS requiring a direct route to invasive coronarography; (4) critical general state; (5) contraindication to the use of regadenoson.

ECG-gated cardiac CT without contrast agent administration for Agatston coronary calcium score measurement was performed in an ambulatory fashion at the Heart and Vascular Centre, Faculty of Medicine, Semmelweis University. In the same setting, patients with a coronary calcium score lower than 400 proceeded to coronary CTA, whereas if the coronary calcium score was higher than 400, regadenoson stress DPCT was performed. Patients identified as having severe coronary artery stenosis by coronary CTA (>70%), and/or perfusion abnormalities involving at least two myocardium segments detected by regadenoson stress DPCT, were referred for invasive coronary angiography as per current standards of care and guidelines [10]. Invasive coronary angiography (ICA) and, if indicated, fractional flow measurements (FFR), percutaneous coronary intervention (PCI), or coronary-artery bypass grafting (CABG) were performed at the respective recruiting institutions at the discretion of the operator in accordance with local protocols [14]. In cases where relevant coronary artery disease as defined above could be ruled out, patients were treated conservatively (optimized medical therapy and lifestyle management).

Diagnosis of hyperlipidemia was based on total cholesterol level >200 mg/dL or the use of lipid-lowering medication. Diabetes mellitus was defined as elevated plasma glucose levels (fasting plasma glucose ≥ 6.5 mmol/L; HbA1C ≥ 6.5%) or the use of antidiabetic medication or insulin therapy. Hypertension was determined as systolic blood pressure > 140 mmHg and/or diastolic blood pressure > 90 mmHg based on in-hospital measurements and necessitating antihypertensive therapy or the prior use thereof. CKD was defined as kidney damage with a glomerular filtration rate (GFR) <60 mL/min/1.73 m^2^ for 3 months or more, irrespective of cause.

The last follow-up visit was performed for all patients by reviewing the patient hospital medical records held in the national health system. Observational time in the study, all-cause death, major adverse cardiovascular events (MACE), and major adverse limb events (MALE) were registered. MACE was defined as stroke, spontaneous myocardial infarction, or cardiovascular death. MALE was defined as untreated loss of patency, reintervention on the index arterial segment, or amputation of the index limb [9,15]. Spontaneous myocardial infarction was defined as Type I, Type II, or Type IVb, according to the Third Universal Definition of Myocardial Infarction [16]. Observational time in the study was defined as the time from index PTA to the end of the study or death.

### 2.2. Cardiac CT Acquisition Protocol

A comprehensive cardiac CT protocol for the evaluation of CAD was performed using a 256-slice multidetector CT scanner (Brilliance iCT, Philips Healthcare, Cleveland, OH, USA) according to the guidelines of the Society of Cardiovascular Computed Tomography [17]. Prospectively ECG-gated non-enhanced CT images of the heart were obtained for coronary calcium scoring using 120 kVp tube voltage with 3 mm slice thickness. The coronary artery calcium score (CACS) was calculated based on the Agatston method from filtered back projection (FBP) images [18].

Afterwards, a prospectively ECG-gated coronary CTA was performed for the evaluation of coronary arteries in cases of <400 CACS. An intravenous beta-blocker was administered before the CTA scan for patients with a heart rate (HR) >65 beats/minute. All patients received 0.8 mg of sublingual nitroglycerine. Image acquisition was conducted during either diastole (at 75–81% of the R–R interval) or systole (at 37–43% of the R–R interval if HR > 75 beats/minute) based on patients’ HR despite premedication. The scan parameters included a gantry rotation time of 270 ms, collimation of 128 × 0.625 mm, tube voltage ranging from 100 to 120 kVp, and tube current adjusted to between 200 and 300 mAs based on the patient’s body mass index. A four-phasic contrast injection protocol was employed, involving the use of 85–95 mL of contrast agent at a flow rate of 4.5–5.5 mL/s [19]. Axial images were reconstructed with a slice thickness of 0.6 mm using iterative reconstruction (iDose4 Level 5, Philips Healthcare, Cleveland, OH, USA). Severe stenosis was defined as a degree of stenosis above 70% in any epicardial coronary artery.

In cases of CACS > 400, a dynamic myocardial perfusion CT scan (DPCT) was performed [20]. For hyperemia induction, a single 400 μg intravenous dose of regadenoson (Rapiscan^®^, GE Healthcare, Chicago, IL, USA) was administered [21]. Stress acquisition was performed during a single breath-hold during inspiration, approximately one minute after administering the bolus of regadenoson during peak stress, covering 25–30 cardiac cycles [22]. The contrast injection protocol included a 50–60 mL contrast bolus at an infusion rate of 5 mL/s, followed by a 30 mL saline chaser. Prospective ECG-gated dynamic-mode imaging was performed (using a 64 × 1.25 mm collimation, 360° reconstruction, and 8 cm coverage) during the systolic phase (at 35% of the R–R interval). Tube voltage settings ranged from 80 to 120 kVp, and tube current was adjusted to between 100 and 250 mAs based on the patient’s BMI. Images were reconstructed using hybrid iterative reconstruction (iDOSE4 Level 5, Philips Healthcare, Cleveland, OH, USA).

The analysis of DPCT images was performed using dedicated software (Intellispace Portal, Philips Healthcare, Cleveland, OH, USA). Time-attenuation curves (TAC) were generated for the left ventricular outflow tract and used as the arterial input function for perfusion analysis. Short-axis views were generated to assess the left ventricular myocardial tissue. Myocardial blood flow (MBF) was calculated using a hybrid deconvolution method [22].

A region of interest (ROI) larger than 0.5 cm^2^ was defined in each myocardial segment (intramural) using a 16-segment model, excluding the apex, with careful attention given to avoiding any artifacts on short-axial images [23]. Myocardial ischemia was defined as MBF less than 101 mL/100 g/min, following the criteria established by Pontone et al. [24].

### 2.3. Integration of Coronary Anatomy and Myocardial Territories

Coronary lesions were assigned to the corresponding myocardial segments based on the modified method after Cerci et al. in the CORE320 (Coronary Artery Evaluation Using 320-Row Multidetector CTA) trial [25]. Vessel-based analysis for the alignment of myocardial territories and supplying large coronary vessels (left (LM), left anterior descending artery (LAD), left circumflex artery (LCX), and right coronary artery (RCA)) was performed by B.S., an expert with 10 years of experience in cardiac imaging [20].

### 2.4. Statistical Analysis

Continuous variables are presented as means with standard deviations, or medians with interquartile ranges, whereas categorical parameters are presented as frequencies with percentages unless otherwise stated. Continuous variables between groups were compared using an independent t-test or Mann–Whitney U test depending on the distribution characteristics of the dataset. Categorical variables were compared using Pearson’s chi-squared test or, in case of 2 × 2 contingency tables, Fisher’s exact test. All statistical analyses were performed using JMP (version 17.0). *p* < 0.05 was defined as statistically significant.

## 3. Results

### 3.1. Study Flowchart

**Figure 1:**
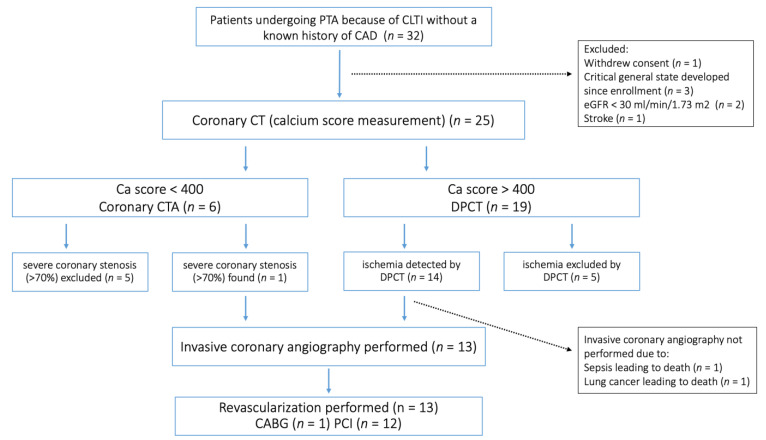
Study flowchart. PTA, percutaneous transluminal angioplasty; CLTI, chronic limb-threatening ischemia; CAD, coronary artery disease; eGFR, estimated glomerular filtration rate; CTA, computer tomography angiography; DPCT, dynamic perfusion computer tomography; CABG, coronary-artery bypass graft surgery; PCI, percutaneous coronary intervention.

Between 2018 and 2023, thirty-two patients with a history of PTA because of CLTI at the three participating institutions were enrolled in the study (Figure 1). Before the planned cardiac CT examination, seven patients were excluded from the study. The median number of days that elapsed between the index PTA and the ambulatory CT examination was 75 days. Of the 25 patients proceeding to calcium score measurement, 6 patients had a calcium score less than 400 and proceeded to coronary CTA, whereas 19 had a calcium score higher than 400, in which cases regadenoson DPCT was performed. In 10 patients, severe coronary artery disease could be ruled out (CTA/DPCT−). Severe coronary artery disease was defined as a diametric stenosis (>70%) on coronary CTA and/or perfusion abnormalities involving at least two myocardium segments detected using regadenoson stress DPCT. Severe coronary artery disease, as defined above, was found in 15 patients (CTA/DPCT+). Coronary angiography was performed in 13 of these patients, prompting revascularization in all 13. In two cases, the planned invasive coronary angiography was not performed due to new-onset severe concomitant disease.

### 3.2. Baseline Clinical and Procedural Characteristics

The baseline clinical characteristics of patients and the procedural details of the index PTA are summarized in Table 1. The prevalence of known risk factors for cardiovascular and lower-extremity PAD was high in the study population: hypertension (88%), hyperlipidemia (92%), smoking (46%), diabetes (52%), and chronic renal disease (24%). Self-reported angina status was low (CCS 0, 1: 90%, 10%) and ejection fraction was preserved (54 ± 6.5%). There were no statistically significant differences between CA/DPCT-positive and -negative patients.

### 3.3. Calcium Score

The overall average Agatston coronary calcium score was high at 1387.58 ± 1751.24, with 19 of the 25 patients having a calcium score higher than 400 and therefore requiring DPCT image acquisition. The Ca score was significantly higher in CTA/DPCT-positive patients than in CTA/DPCT-negative patients (2188.5 ± 1939.19 versus 266.3 ± 235.72; *p* < 0.001).

### 3.4. Comparison of CTA/DPCT and Invasive Coronary Angiography

In all 13 patients proceeding to coronary angiography as planned, invasive evaluation verified the previously CTA/DPCT-imaging-predicted severe coronary artery disease (Figure 1). Overall, the per-patient positive predictive value for revascularization in this special subset for CTA/DPCT imaging was 100%.

To further characterize the value of coronary CTA/DPCT imaging, a vessel-based comparison with ICA was also performed in this patient subset. Severe CAD was defined as a diametric stenosis >90% and/or an FFR value < 0.8 during CAG, prompting revascularization. Alignment of perfusion defects to large coronary vessels was performed as described above. Fifty-two large vessels of the 13 patients were analyzed. Severe CAD was found in 25/52 (48%) large coronary vessels using invasive evaluation and using CTA/DPCT imaging. Vessel-based comparison results between coronary CTA/DPCT and invasive angiography were as follows: sensitivity 75%, specificity 75%, positive predictive value 72%, and negative predictive value 78%.

### 3.5. Follow-Up

The average time of follow-up in this prospective registry was 27 ± 21 months. The rates of major adverse events overall were as follows: all-cause death 8/25, 32%; MACE 4/25, 12%; MALE 6/25, 24%. There were no statistically significant differences as regards to follow-up endpoints between CTA/DPCT− and CTA/DPCT+ patient groups. However, a clear tendency towards higher rates of all-cause death (7/15, 45% versus 1/10, 10%; *p* = 0.065) and MACE (4/15, 27% versus 0/10, 0%; *p* = 0.11) in the CTA/DPCT+ patients could be seen (Table 2).

## 4. Discussion

To the best of our knowledge, we believe this is the first study suggesting that the combination of coronary CTA with stress DPCT is useful in the detection of functionally significant obstructive CAD in CLTI patients.

Chronic limb-threatening ischemia, which is at the end stage of the peripheral artery disease spectrum, is associated with excessively high risk for cardiovascular events, myocardial infarction, and death [2,5,6,7,26]. Atherosclerosis is the underlying pathophysiological connection between peripheral and coronary artery disease. CAD and PAD share several common risk factors for development of atherosclerosis, such as smoking, dyslipidemia, hypertension, and diabetes mellitus [26]. Atherosclerosis, formerly thought to be caused primarily by dyslipidemia and lipid accumulation in the endothelial wall, is today viewed more as an inflammatory process [27,28,29]. Indeed, the widespread activation of inflammatory cells across the vascular bed connects different anatomical manifestations of atherosclerosis [28]. Despite the common pathophysiology and shared risk factors, differences in manifestations of atherosclerosis in PAD and CAD have to be noted. For example, lipid-rich fibroatheromatic plaques are more common in CAD [30], whereas in PAD, common histological findings include fibroproliferative plaques with low lipid content and a high vascular smooth cell content [30,31]. These findings explain why PAD is a more stable form of atherosclerotic disease as compared to CAD. Acute events in PAD are more often linked to embolization or in situ thrombosis in contrast to the plaque rupture and atherothrombosis that occurs in CAD [28].

The severity of PAD is directly associated with the likelihood of concomitant CAD, with up to 90% of patients presenting with CLTI also having CAD. The high prevalence of CAD in patients with CLTI should clearly underline the importance of diagnosis of obstructive CAD to minimize the risk of cardiac events. However, to date, systematic screening for CAD and aggressive revascularization-driven treatment approaches have provided no improvement in cardiovascular clinical outcomes for PAD or CLTI patients [32,33]. Accordingly, current guidelines do not advise systematic screening for CAD in the general PAD population, irrespective of anginiform symptoms [34,35,36,37].

**Figure 2:**
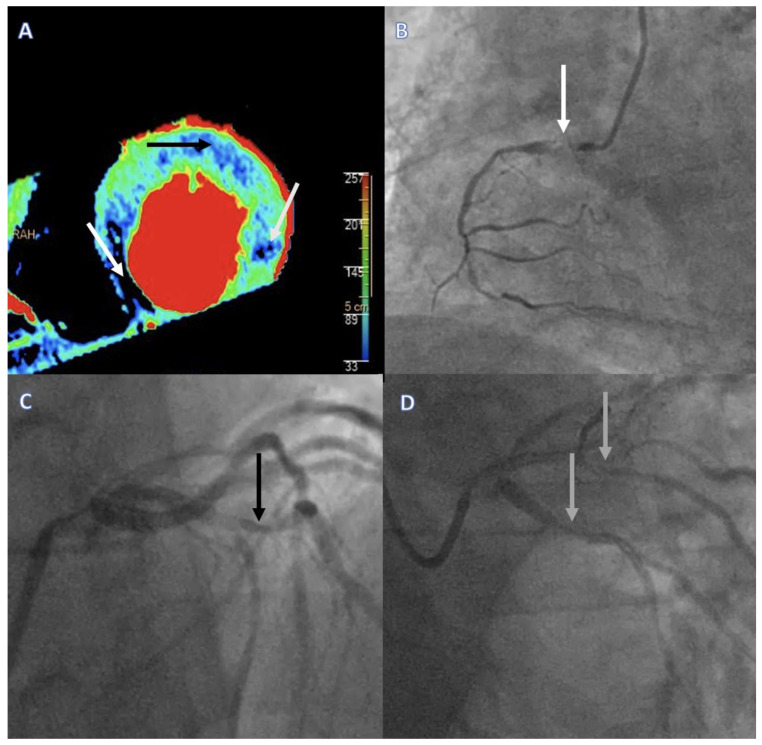
Regadenoson stress DPCT imaging results and corresponding invasive angiography and FFR findings in a multivessel CAD patient. (**A**) Regadenoson stress DPCT image depicting reduced myocardial blood flow (MBF < 101 mL/100 g/min) for all three main coronary territories. Necrosis was detected in the inferoseptal region (MBF 36 mL/100 g/min, white arrow), whereas ischemia was provoked in the anterior (MBF 69 mL/100 g/min, black arrow) and lateral (MBF 84 mL/100 g/min, gray arrow) wall of the LV. (**B**–**D**) Corresponding invasive coronary angiography images verifying a chronic total occlusion of the right coronary artery (white arrow), severe stenosis of the left anterior descending artery (FFR = 0.65) (black arrow), and severe stenosis of the circumflex (FFR = 0.75) and intermediary coronary arteries (gray arrows). DPCT = dynamic perfusion computed tomography, FFR = fractional flow reserve, MBF = myocardial blood flow, LV = left ventricle.

The combination of anatomical and functional imaging by coronary CTA/regadenoson stress DPCT has several advantages in comparison to standard approaches to obstructive CAD screening in CLTI patients: (1) unnecessary invasive examinations in patients where peripheral access/re-access for catheterization is difficult may be avoided, and invasive coronary angiography could be restricted to patients where revascularization is probable [38]; (2) anatomical evaluation with pharmacological stress testing is ideal for detecting silent ischemia in the mobility-impaired CLTI population; and (3) DPCT utilizing absolute values of myocardial blood flow is more precise than SPECT relying on the differences between normal and ischemic myocardium and, thus, is better in the frequent setting of left main or multivessel disease in this patient population (Figure 2) [39] However, some limitations of this method must also be noted: (1) contraindication to regadenoson; (2) contraindication due to impaired renal function; and (3) contraindication due to poor general state and/or lack of cooperation, which are also more frequent in the CLTI patient population.

The Agatston coronary calcium score in CLTI patients was high, above 1300 on average, with 19 of the 25 patients (76%) having a calcium score higher than 400. Our findings coincide with the results of Konijn et al., who reported 57% of CLTI patients having a very high coronary calcium score (>1000) [40]. Sixty percent of our CLTI patients were found by coronary CTA/DPCT to have severe CAD. These findings are similar to those reported by Lee et al., who performed coronary angiography in 252 consecutive patients with a history of lower-limb angioplasty for critical limb ischemia and found angiographically significant CAD in 145 patients (57.5%) [32]. The positive predictive value of coronary CTA/DPCT, justifying the need for revascularization, was 100% on a per-patient level. These findings support the notion of coronary CTA/DPCT as a possible gatekeeper to ICA and revascularization. On a per-vessel basis, efficacy of coronary CTA/DPCT was modest as compared to ICA and, if necessary, FFR, with 75% sensitivity and specificity, 72% positive predictive value, and 78% negative predictive value. In a recent multicenter trial using third-generation dual-source scanners, DPCT showed a sensitivity of (84%; 95% CI: 75–92%) and higher specificity (89%; 95% CI: 85–93%) as compared to ICA and FFR. However, these results were obtained by investigating a “healthier” population, with 26% of vessels affected in comparison to 48% in the current study [41].

The rate of major adverse events at an average follow-up time of 27 months (all-cause death 32%, MACE 12%, and MALE 24%) was similar to previously published results [3,4,5,6,7,15]. Risk stratification using our approach seems to be feasible, as there was a clear tendency towards higher all-cause death and MACE in coronary CTA/DPCT-positive versus -negative CLTI patients (Table 2). A prospective comparative study of clinical outcomes, comparing conservative management and a CTA/DCTP imaging-guided group, could further clarify the clinical relevance of such a risk stratification strategy.

Several limitations of this study must be noted. First, due to the COVID-19 pandemic, the number of patients enrolled in the study was low, resulting in the study’s pilot nature. Thus, the study was underpowered for a meaningful analysis of Kaplan–Meier survival and MACE/MALE-free survival curves, both in the overall population and in the comparison CTA/DPCT-positive and -negative groups. Secondly, as patient pathways were clinically driven, not all patients proceeded to invasive coronary angiography and FFR. Thus, a formal comparison for sensitivity and specificity of DCTP and invasive coronary angiography/FFR results was not possible overall. Thirdly, as only patients without prior history of severe cardiac disease and who underwent successful PTA procedure were enrolled in the study, results may not be extrapolated to the entire CLTI patient population.

## 5. Conclusions

Coronary CTA complemented by regadenoson stress DPCT in cases of severe coronary artery calcification can be a reliable non-invasive method to detect obstructive and functionally significant CAD in CLTI patients. Further studies are warranted in larger populations to assess whether risk stratification based on this strategy can lead to a reduction in otherwise high cardiovascular events in this patient population.

## Figures and Tables

**Table 1:** Clinical characteristics of patients and procedural details of index PTA.

Parameter	All Patients (*n* = 25)	CTA/DPCT− (*n* = 10)	CTA/DPCT+ (*n* = 15)	*p* Value
Demography				
Gender (male)	18 (69)	6 (60)	12 (75)	0.66
Age (years)	64.4 ± 8.7	63 ± 10.1	65.3 ± 7.9	0.53
Medical history				
Smoker or former smoker	12 (48)	3 (30)	9 (60)	0.14
Hypertension	22 (88)	7 (70)	15 (100)	0.052
Diabetes mellitus	13 (52)	3 (30)	10 (67)	0.08
CKD	6 (24)	3 (30)	3 (60)	0.45
Hyperlipidemia	23 (92)	8 (80)	15 (100)	0.15
Cardiac evaluation				
CCS angina grade (0–4)				0.46
0	21 (84)	9 (90)	12 (81)	
1	4 (16)	1 (10)	3 (19)	
EF (%)	54 ± 6.5	56 ± 5.4	52.6 ± 7	0.12
CLTI severity, Rutherford classification (1–6)				0.69
4	15 (60)	6 (60)	9 (60)	
5	9 (36)	4 (40)	5 (33)	
6	1 (4)	0	1 (7)	
Procedural data of PTA (localization)				0.71
Iliac artery	4 (16)	1 (10)	3 (20)	
SFA	8 (32)	3 (30)	5 (33)	
BTK	7 (28)	2 (20)	5 (33)	
SFA/iliac artery + BTK	6 (24)	4 (40)	2 (13)	

CTA/DPCT−: patients in whom severe coronary artery disease was ruled out by coronary CTA/DCTP; CTA/PCT+: patients in whom severe coronary artery disease was suspected by coronary CTA/DCTP; BTK, below the knee; CAD, coronary artery disease; CCS, Canadian Cardiovascular Society; CKD, chronic kidney disease; CLTI, chronic limb-threatening ischemia; CTA, computer tomography angiography; DPCT, dynamic perfusion computed tomography; (N)OAC novel oral anticoagulant; PTA, percutaneous transluminal angioplasty; SFA, superficial femoral artery.

**Table 2:** Results of follow-up.

Parameter	All Patients (*n* = 25)	CTA/DPCT− (*n* = 10)	CTA/DPCT+ (*n* = 15)	*p* Value
Observational time (months)	27 ± 21	33 ± 23	23 ± 19	0.38
All-cause death	8 (32)	1 (10)	7 (47)	0.065
MACE	4 (16) #	0	4 (27)	0.11
spontaneous MI	3 (12) *	0	3 (20)	0.20
stroke	0	0	0	N. A
CV death	3 (12)	0	3 (20)	0.20
MALE	7 (28)	3 (30)	4 (27)	0.86
untreated loss of patency	2 (8)	0	2 (13)	0.35
amputation	1 (4)	1 (10)	0	0.21
reintervention	4 (16)	2 (20)	2 (13)	0.84

CTA/DPCT−: patients in whom severe coronary artery disease was ruled out by coronary CTA/DCTP; CTA/DPCT+: patients in whom severe coronary artery disease was suspected by coronary CTA/DCTP; **#** rows in MACE do not add up as two patients had two events; * two of the three patients had a Type IVb MI caused by angiographically verified stent thrombosis; MACE, major adverse cardiac events; MALE, major adverse limb events; MI, myocardial infarction; CTA, computer tomography angiography; DPCT, dynamic perfusion computed tomography.

## Data Availability

Data supporting the findings of this study are available from the corresponding author, upon reasonable request.

## References

[1] Criqui M.H., Matsushita K., Aboyans V., Hess C.N., Hicks C.W., Kwan T.W., McDermott M.M., Misra S., Ujueta F. (2021). Lower Extremity Peripheral Artery Disease: Contemporary Epidemiology, Management Gaps, and Future Directions: A Scientific Statement From the American Heart Association. Circulation.

[2] Farber A. (2018). Chronic Limb-Threatening Ischemia. N. Engl. J. Med..

[3] Farber A., Menard M.T., Conte M.S., Kaufman J.A., Powell R.J., Choudhry N.K., Hamza T.H., Assmann S.F., Creager M.A., Cziraky M.J. (2022). Surgery or Endovascular Therapy for Chronic Limb-Threatening Ischemia. N. Engl. J. Med..

[4] Soga Y. (2021). Two-year Mortality in CLTI Patients Provide Crucial Factors We Should Fight. J. Atheroscler. Thromb..

[5] Simons J.P., Schanzer A., Flahive J.M., Osborne N.H., Mills J.L., Bradbury A.W., Conte M.S. (2019). Survival prediction in patients with chronic limb-threatening ischemia who undergo infrainguinal revascularization. J. Vasc. Surg..

[6] Bradbury A.W., Adam D.J., Bell J., Forbes J.F., Fowkes F.G., Gillespie I., Ruckley C.V., Raab G.M. (2010). Bypass versus Angioplasty in Severe Ischaemia of the Leg (BASIL) trial: An intention-to-treat analysis of amputation-free and overall survival in patients randomized to a bypass surgery-first or a balloon angioplasty-first revascularization strategy. J. Vasc. Surg..

[7] Hata Y., Iida O., Asai M., Masuda M., Okamoto S., Ishihara T., Nanto K., Kanda T., Tsujumura T., Okuno S. (2021). Risk Stratification for 2-Year Mortality in Patients with Chronic Limb-Threatening Ischemia Undergoing Endovascular Therapy. J. Atheroscler. Thromb..

[8] Criqui M.H., Langer R.D., Fronek A., Feigelson H.S., Klauber M.R., McCann T.J., Browner D. (1992). Mortality over a period of 10 years in patients with peripheral arterial disease. N. Engl. J. Med..

[9] Toth G., Brodmann M., Barbato E., Mangiacapra F., Schneller L., Orias V., Gil R., Bil J., Bartus S., Ruzsa Z. (2019). Rational and design of the INtentional COronary revascularization versus conservative therapy in patients undergOing successful peripheRAl arTEry revascularization due to critical limb ischemia trial (INCORPORATE trial). Am. Heart J..

[10] Knuuti J., Wijns W., Saraste A., Capodanno D., Barbato E., Funck-Brentano C., Prescott E., Storey R.F., Deaton C., Cuisset T. (2020). 2019 ESC Guidelines for the diagnosis and management of chronic coronary syndromes. Eur. Heart J..

[11] Kwan A.C., Gransar H., Tzolos E., Chen B., Otaki Y., Klein E., Pope A.J., Han D., Howarth A., Jain N. (2021). The accuracy of coronary CT angiography in patients with coronary calcium score above 1000 Agatston Units: Comparison with quantitative coronary angiography. J. Cardiovasc. Comput. Tomogr..

[12] Nieman K., Balla S. (2020). Dynamic CT myocardial perfusion imaging. J. Cardiovasc. Comput. Tomogr..

[13] Conte M.S., Bradbury A.W., Kolh P., White J.V., Dick F., Fitridge R., Mills J.L., Ricco J.B., Suresh K.R., Murad M.H. (2019). Global vascular guidelines on the management of chronic limb-threatening ischemia. J. Vasc. Surg..

[14] Neumann F.J., Sousa-Uva M., Ahlsson A., Alfonso F., Banning A.P., Benedetto U., Byrne R.A., Collet J.P., Falk V., Head S.J. (2019). 2018 ESC/EACTS Guidelines on myocardial revascularization. Eur. Heart J..

[15] Fashandi A.Z., Mehaffey J.H., Hawkins R.B., Kron I.L., Upchurch G.R., Robinson W.P. (2018). Major adverse limb events and major adverse cardiac events after contemporary lower extremity bypass and infrainguinal endovascular intervention in patients with claudication. J. Vasc. Surg..

[16] Thygesen K., Alpert J.S., Jaffe A.S., Simoons M.L., Chaitman B.R., White H.D., Thygesen K., Alpert J.S., White H.D., Jaffe A.S. (2012). Third universal definition of myocardial infarction. Eur. Heart J..

[17] Abbara S., Blanke P., Maroules C.D., Cheezum M., Choi A.D., Han B.K., Marwan M., Naoum C., Norgaard B.L., Rubinshtein R. (2016). SCCT guidelines for the performance and acquisition of coronary computed tomographic angiography: A report of the society of Cardiovascular Computed Tomography Guidelines Committee: Endorsed by the North American Society for Cardiovascular Imaging (NASCI). J. Cardiovasc. Comput. Tomogr..

[18] Agatston A.S., Janowitz W.R., Hildner F.J., Zusmer N.R., Viamonte M., Detrano R. (1990). Quantification of coronary artery calcium using ultrafast computed tomography. J. Am. Coll. Cardiol..

[19] Karády J., Panajotu A., Kolossváry M., Szilveszter B., Jermendy Á.L., Bartykowszki A., Károlyi M., Celeng C., Merkely B., Maurovich-Horvat P. (2017). The effect of four-phasic versus three-phasic contrast media injection protocols on extravasation rate in coronary CT angiography: A randomized controlled trial. Eur. Radiol..

[20] Vattay B., Boussoussou M., Borzsák S., Vecsey-Nagy M., Simon J., Kolossváry M., Merkely B., Szilveszter B. (2021). Myocardial perfusion imaging using computed tomography: Current status, clinical value and prognostic implications. Imaging.

[21] Iskandrian A.E., Bateman T.M., Belardinelli L., Blackburn B., Cerqueira M.D., Hendel R.C., Lieu H., Mahmarian J.J., Olmsted A., Underwood S.R. (2007). Adenosine versus regadenoson comparative evaluation in myocardial perfusion imaging: Results of the ADVANCE phase 3 multicenter international trial. J. Nucl. Cardiol..

[22] Tanabe Y., Kido T., Uetani T., Kurata A., Kono T., Ogimoto A., Miyagawa M., Soma T., Murase K., Iwaki H. (2016). Differentiation of myocardial ischemia and infarction assessed by dynamic computed tomography perfusion imaging and comparison with cardiac magnetic resonance and single-photon emission computed tomography. Eur. Radiol..

[23] Vattay B., Borzsák S., Boussoussou M., Vecsey-Nagy M., Jermendy Á.L., Suhai F.I., Maurovich-Horvat P., Merkely B., Kolossváry M., Szilveszter B. (2022). Association between coronary plaque volume and myocardial ischemia detected by dynamic perfusion CT imaging. Front. Cardiovasc. Med..

[24] Pontone G., Baggiano A., Andreini D., Guaricci A.I., Guglielmo M., Muscogiuri G., Fusini L., Soldi M., Del Torto A., Mushtaq S. (2019). Dynamic Stress Computed Tomography Perfusion with a Whole-Heart Coverage Scanner in Addition to Coronary Computed Tomography Angiography and Fractional Flow Reserve Computed Tomography Derived. JACC Cardiovasc. Imaging.

[25] Cerci R.J., Arbab-Zadeh A., George R.T., Miller J.M., Vavere A.L., Mehra V., Yoneyama K., Texter J., Foster C., Guo W. (2012). Aligning coronary anatomy and myocardial perfusion territories: An algorithm for the CORE320 multicenter study. Circ. Cardiovasc. Imaging.

[26] Aday A.W., Matsushita K. (2021). Epidemiology of Peripheral Artery Disease and Polyvascular Disease. Circ. Res..

[27] Soehnlein O., Libby P. (2021). Targeting inflammation in atherosclerosis—From experimental insights to the clinic. Nat. Rev. Drug Discov..

[28] Achim A., Péter O., Cocoi M., Serban A., Mot S., Dadarlat-Pop A., Nemes A., Ruzsa Z. (2023). Correlation between Coronary Artery Disease with Other Arterial Systems: Similar, Albeit Separate, Underlying Pathophysiologic Mechanisms. J. Cardiovasc. Dev. Dis..

[29] Poledniczek M., Neumayer C., Kopp C.W., Schlager O., Gremmel T., Jozkowicz A., Gschwandtner M.E., Koppensteiner R., Wadowski P.P. (2023). Micro- and Macrovascular Effects of Inflammation in Peripheral Artery Disease-Pathophysiology and Translational Therapeutic Approaches. Biomedicines.

[30] Matsuo Y., Takumi T., Mathew V., Chung W.Y., Barsness G.W., Rihal C.S., Gulati R., McCue E.T., Holmes D.R., Eeckhout E. (2012). Plaque characteristics and arterial remodeling in coronary and peripheral arterial systems. Atherosclerosis.

[31] Poredos P., Poredos P., Jezovnik M.K. (2018). Structure of Atherosclerotic Plaques in Different Vascular Territories: Clinical Relevance. Curr. Vasc. Pharmacol..

[32] Lee M.S., Rha S.W., Han S.K., Choi B.G., Choi S.Y., Park Y., Akkala R., Li H., Im S.I., Kim J.B. (2015). Clinical outcomes of patients with critical limb ischemia who undergo routine coronary angiography and subsequent percutaneous coronary intervention. J. Invasive Cardiol..

[33] McFalls E.O., Ward H.B., Moritz T.E., Goldman S., Krupski W.C., Littooy F., Pierpont G., Santilli S., Rapp J., Hattler B. (2004). Coronary-artery revascularization before elective major vascular surgery. N. Engl. J. Med..

[34] Halliday A., Bax J.J. (2018). The 2017 ESC Guidelines on the Diagnosis and Treatment of Peripheral Arterial Diseases, in Collaboration With the European Society for Vascular Surgery (ESVS). Eur. J. Vasc. Endovasc. Surg..

[35] Abramson B.L., Al-Omran M., Anand S.S., Albalawi Z., Coutinho T., de Mestral C., Dubois L., Gill H.L., Greco E., Guzman R. (2022). Canadian Cardiovascular Society 2022 Guidelines for Peripheral Arterial Disease. Can. J. Cardiol..

[36] Aboyans V., Ricco J.B., Bartelink M.E.L., Björck M., Brodmann M., Cohnert T., Collet J.P., Czerny M., De Carlo M., Debus S. (2018). 2017 ESC Guidelines on the Diagnosis and Treatment of Peripheral Arterial Diseases, in collaboration with the European Society for Vascular Surgery (ESVS): Document covering atherosclerotic disease of extracranial carotid and vertebral, mesenteric, renal, upper and lower extremity arteriesEndorsed by: The European Stroke Organization (ESO)The Task Force for the Diagnosis and Treatment of Peripheral Arterial Diseases of the European Society of Cardiology (ESC) and of the European Society for Vascular Surgery (ESVS). Eur. Heart J..

[37] Frank U., Nikol S., Belch J., Boc V., Brodmann M., Carpentier P.H., Chraim A., Canning C., Dimakakos E., Gottsäter A. (2019). ESVM Guideline on peripheral arterial disease. Vasa.

[38] Sandoval Y., Burke M.N., Lobo A.S., Lips D.L., Seto A.H., Chavez I., Sorajja P., Abu-Fadel M.S., Wang Y., Poulouse A. (2017). Contemporary Arterial Access in the Cardiac Catheterization Laboratory. JACC Cardiovasc. Interv..

[39] Afonso L., Mahajan N. (2009). Single-photon emission computed tomography myocardial perfusion imaging in the diagnosis of left main disease. Clin. Cardiol..

[40] Konijn L.C.D., Mali W., van Overhagen H., Takx R.A.P., Veger H.T.C., de Jong P.A. (2023). Systemic arterial calcium burden in patients with chronic limb-threatening ischemia. J. Cardiovasc. Comput. Tomogr..

[41] Nous F.M.A., Geisler T., Kruk M.B.P., Alkadhi H., Kitagawa K., Vliegenthart R., Hell M.M., Hausleiter J., Nguyen P.K., Budde R.P.J. (2022). Dynamic Myocardial Perfusion CT for the Detection of Hemodynamically Significant Coronary Artery Disease. JACC Cardiovasc. Imaging.

